# Isolation, Identification, and Complete Genome Assembly of an Endophytic *Bacillus velezensis* YB-130, Potential Biocontrol Agent Against *Fusarium graminearum*

**DOI:** 10.3389/fmicb.2020.598285

**Published:** 2020-12-03

**Authors:** Wen Xu, Liyong Zhang, Paul H. Goodwin, Mingcong Xia, Jie Zhang, Qi Wang, Juan Liang, Runhong Sun, Chao Wu, Lirong Yang

**Affiliations:** ^1^Institute of Plant Protection Research, Henan Academy of Agricultural Sciences, Henan Biopesticide Engineering Research Center, Henan International Joint Laboratory of Crop Protection, Zhengzhou, China; ^2^College of Life Sciences, Henan Agricultural University, Zhengzhou, China; ^3^School of Environmental Sciences, University of Guelph, Guelph, ON, Canada; ^4^College of Plant Protection, China Agricultural University, Beijing, China

**Keywords:** *Bacillus velezensis*, *Fusarium graminearum*, DON, biocontrol agent, genome sequencing and assembly

## Abstract

Wheat scab caused by *F. graminearum* is a highly destructive disease that leads to yield reduction and mycotoxin contamination of grains. In this study, an endophytic bacterium of strain YB-130 was isolated from surface sterilized wheat spikes with scab symptoms and identified as *Bacillus velezensis* by whole genome annotation, 16S rRNA gene and average nucleotide identities analysis. The whole-genome sequence of strain YB-130 was obtained by PacBio sequencing. 88 putative Carbohydrate-Active Enzymes and 12 gene clusters encoding for secondary metabolites were identified in the YB-130 genome, including one gene cluster for the synthesis of lanthipeptide only found in strain YB-130 genome. In dual cultures, strain YB-130 significantly inhibited the growth of *F. graminearum* PH-1 and other eight fungal plant pathogens, indicating a broad antifungal activity. Furthermore, strain YB-130 was able to significantly inhibit spore morphology and hyphal development of *F. graminearum* PH-1. Strain YB-130 also reduced deoxynivalenol production by *F. graminearum* PH-1 in dual cultures, possibly due to its ability to suppress the expression of *tri5*, *tri3*, and *tri8* that are required for deoxynivalenol production in *F. graminearum*. Overall, *B. velezensis* YB-130 is a promising biological control agent of both *F. graminearum* infection and mycotoxin production.

## Introduction

Wheat (*Triticum aestivum* L.) is a major staple food crop for human and livestock worldwide (Shewry, 2009). Wheat production and grain quality is affected by multiple fungal diseases (Figueroa et al., 2018). Of these, wheat scab caused by *F. graminearum* is considered to be one of most devastating diseases of wheat worldwide, not only because it leads to severe yield losses but also because infected grains contain harmful trichothecene mycotoxins, such as deoxynivalenol (DON), that pose serious threats to food safety and human health (McMullen et al., 1997; Goswami and Kistler, 2006; Chen et al., 2019; Savary et al., 2019). So far, 14 different *tri* genes have been identified for DON biosynthesis in *F. graminearum* (Chen et al., 2019). Among them are *tri5* encoding a trichodiene synthase that cyclizes farnesyl pyrophosphate to trichodiene in the first step of DON biosynthesis, *tri3* encoding a calonectrin synthase that acetylates 15-decalonectrin to form calonectrin, and *tri8* encoding a trichothecene C-3 esterase that catalyzes the deacetylation of 3,15-diacetyldeoxynivalenol to 3-ADON or 15-ADON followed by DON in the final step of DON biosynthesis (McCormick et al., 1996; McCormick and Alexander, 2002).

During the past few decades, common approaches to controlling wheat scab have included the development of resistant cultivars and the use of chemical pesticides (Parry et al., 1995; Yuen and Schoneweis, 2007; Nelson et al., 2018; Pan et al., 2019). However, there are only a few commercially available resistant cultivars to protect against wheat scab, and the overuse of fungicides against wheat scab has resulted in fungicide resistance, environmental damage and potential health hazards in humans and animals (McAleese, 1971; Kim et al., 2017; Fisher et al., 2018). Biological control by microorganisms to suppress plant pathogens is an appealing alternative.

There have been a number of reports of using endophytic *Bacillus* species for biological control of wheat scab. For example, endopyhtic *Bacillus megaterium* and *Bacillus subtilis* isolated from wheat grain significantly reduced fungal growth and spore germination of *F. graminearum* (Pan et al., 2015). An endophytic *Bacillus atrophaeus* XM5 isolated from wheat leaves was able to strongly inhibit *Fusarium* head blight by *F. graminearum* and the control efficiency reached 68.3% after 7 days (Xin et al., 2013). Inoculation of wheat kernels with *Bacillus mojavensis* RRC 101 from maize kernels reduced seedling blight of wheat produced by *F. graminearum* and increased seedling emergence from 20 to 82% (Bacon and Hinton, 2007). *Bacillus subtilis* SG6 from wheat kernels strongly inhibited mycelium growth, sporulation and DON production of *F. graminearum* (Zhao et al., 2014). In summary, endopyhtic *Bacillus* species appear to hold considerable potential as biological control agents for wheat scab.

*Bacillus* species can be so effective in inhibiting plant pathogens because they produce many antimicrobial compounds, such as hydrolases and secondary metabolites, and as a result, this has lead to the isolation and identification of a large number of potential plant disease biocontrol strains (Caulier et al., 2019). Part of the confusion as to which species have been effective has been due to identification of *Bacillus* species based on single gene sequence, such as the 16S rRNA gene (Dunlap, 2019). However, classifications based on whole-genome sequences and analysis, such as by average nucleotide identities (ANI), allow for better identication of isolates as well as better demarcation of the taxonomic relationship among some closely related *Bacillus* species (Baek et al., 2019; Liu et al., 2019). Whole-genome sequence and annotation also allows for the discovery of genes related to factors that contribute to biocontrol activity, such as genes for Carbohydrate-Active Enzymes (CAZymes) and production of various secondary metabolites that act as mechanisms of inhibition of hyphal development and DON production by *F. graminearum*.

In this study, strain YB-130 was isolated from surface sterilized spikes of wheat with scab symptoms and identified as *Bacillus velezensis* by morphological and molecular approaches. In addition, the genome sequence of strain YB-130 was sequenced, and genes for CAZymes and secondary metabolites were identified. In dual culture, it exhibited strong antifungal activity against *F. graminearum* PH-1 and eight other fungal plant pathogens, and it also reduced the production of DON by *F. graminearum* PH-1, which correlated with decreased expression levels of three DON synthesis-related genes, *tri5*, *tri3* and *tri8*. These results indicated that *B. velezensis* YB-130 may be a potential biocontrol agent for controlling wheat scab disease and DON production caused by *F. graminearum*.

## Materials and Methods

### Wheat Sample Collection

*Triticum aestivum* cv. Zhengmai 366 was grown in the field at the Xihua County Agricultural Science Institute, Henan Province, China. Spikes of wheat scab were collected in a sterile polythene bag and transferred immediately to the laboratory for the isolation of endophytic bacteria.

### Bacteria and Pathogens Used in This Study

YB-130 was isolated and screened from spikes of wheat scab. Strains of *F. graminearum* PH-1, *Gaeumannomyces graminis var. tritici*, *Rhizoctonia cerealis*, *Bipolaris sorokiniana, Fusarium oxysporum f.sp. niveum*, *Fusarium oxysporum f.sp. capsicum*, *Fusarium oxysporum f.sp. cucumebrium*, *Pythium myriotylum*, and *Cercospora apii* were obtained from the fungal culture collection of the Institute of Plant Protection Research, Henan Academy of Agricultural Sciences, Zhengzhou, China.

### Isolation and Morphological Observation of Endophytic Bacteria

For bacterial isolation, glumes, lemmas and paleas of wheat spikes were separated, and then washed for 5 min with sterile distilled water. The tissues were surface sterilized with 75% ethanol for 2–3 min, 3% sodium hypochlorite for 4–5 min followed by rinsing twice with sterile distilled water. Then, the samples were dried on sterile filter paper and homogenized with a sterile mortar and pestle. After grinding, 10 ml sterile water was added to the mortar, and 10 μl of homogenate was transferred to nutrient agar (NA) in triplicate and incubated at 28°C for 2–4 days. As a control, 10 μl of the last wash water was plated. Single colonies were transferred to LB agar media to obtain single colonies for pure cultures. After strain YB-130 was grown for 24 h on LB agar medium at 28°C, colony color and shape was recorded as well as the results of Gram staining performed as per (Coico, 2005). The same colonies were observed with scanning electron microscopy (SEM) using a Hitachi SU8100 microscopy (Hitachi Higher Technologies Corp., Tokyo, Japan).

### DNA Extraction, Genome Sequencing and Assembly of Strain YB-130

A single colony of bacterial strain YB-30 was inoculated in Nutrient Broth and grown for 18 h at 28°C shaking at 150 rpm. Genomic DNA of YB-130 was extracted by the Mini-BEST Bacterial Genomic DNA Extraction Kit Ver. 3.0 (Takara, Beijing, China) following the manufacturer’s instructions. An approximately 10 Kb insert PacBio library was constructed, and whole genome sequencing was performed with the PacBio Sequel II system (Pacific Biosciences, Menlo Park, CA, United States). The PacBio reads were *de novo* assembled using the HGAP4 (Chin et al., 2013) and Canu (v.1.6) (Koren et al., 2017) software. The depth of genome coverage were analyzed by pbalign (BLASR, v0.4.1) tool (Chaisson and Tesler, 2012). The assembled genome sequence of strain YB-130 has been deposited in NCBI GenBank with the accession number CP054562. A circular map of strain YB-130 genome were obtained with Circos (v0.64) (Krzywinski et al., 2009).

### RNA Sequencing and Mapping Reads to the Genome of Strain YB-130

RNA sequencing was done from pure cultures of strain YB-130 grown for 24 h in NB broth at 28°C shaking at 150 rpm. Total RNA was extracted using the RNAprep Pure Cell/Bacteria Kit (Tiangen, Beijing, China). Sequencing libraries were generated using NEBNext Ultra RNA Library Prep Kit for Illumina (NEB, Beverly, MA, United States) following manufacturer’s instructions. Paired-end 150 bp reads were sequenced on an Illumina NovaSeq sequencing system (Illumina, San Diego, CA, United States). The sequencing reads were mapped to the assembled genome sequence with the program Bowtie2 (v2.2.3) (Langmead and Salzberg, 2012).

### Genome Annotation of Strain YB-130

The genome of strain YB-130 was annotated using Glimmer (v3.02) (Delcher et al., 2007). The tRNA and rRNA genes were identified by tRNAscan-SE (v2.0) (Lowe and Eddy, 1997) and RNAmmer (v1.2) (Lagesen et al., 2007), respectively. The functional characterization of the identified genes was achieved by BLASTx with an *E*-value threshold of 1e-5 against the databases of the NCBI Non-Redundant protein database (NR), Swiss-Prot, Clusters of Orthologous Groups (COG), Kyoto Encyclopedia of Genes and Genomes (KEGG), and Gene Ontology (GO). The match with the highest score was considered to be as the final annotation of one specific gene.

### Molecular Identification of Strain YB-130

The 16S rRNA gene sequences of strain YB-130 and other 11 *Bacillus* species (*B. velezensis* FZB42, *B. velezensis* FJAT-45028, *B. velezensis* CAU B946, *B. subtilis* H1, *B. subtilis* 168, *B. licheniformis* SRCM103583, *B. licheniformis* ATCC 14580, *B. altitudinis* CHB19, *B. altitudinis* GQYP101, *B. pumilus* SF-4 and *B. pumilus* ZB201701) were identified from the complete genome sequences (GenBank IDs: CP000560.2, CP047157.1, HE617159.1, CP026662.1, NC_000964.3, CP035404.1, CP034569.1, CP043559.1, CP040514.1, CP047089.1, and CP029464.1), respectively. A phylogenetic tree of the 16S rRNA gene sequences were constructed with MEGA 7.0 using the Neighbour Joining method (Kumar et al., 2016). Average Nucleotide Identity (ANI) between strain YB-130 and 11 *Bacillus* strains with complete genome sequences was calculated using ANI calculator (Yoon et al., 2017).

### Analysis of CAZymes and Secondary Metabolic Genes of Strain YB-130

Predicted protein sequences from the genome of strain YB-130 were aligned with the carbohydrate active enzyme (CAZy) database^1^ (Lombard et al., 2014) using dbCAN2 (Zhang et al., 2018) and HMMER (v3.1b2) (Finn et al., 2011) with an *E*-value threshold of 1e-15. Signal peptides of identified CAZymes were analyzed by SignalP (v4.1) (Petersen et al., 2011). Identification of gene clusters for secondary metabolite synthesis was analyzed by antiSMASH^2^ (Blin et al., 2019).

Strain YB-130 were grown in 600 ml LB medium at 37°C with shaking at 200 rpm for 40 h. Strain YB-130 cells were removed from the surfactin-containing medium by centrifugation (10000 rpm for 10 min at 4°C). Extraction of lipopeptides from the supernatant was carried out according to the methods and analyzed by Matrix-assisted laser desorption ionization time-of-flight tandem mass spectrometry (MALDI-TOF-MS) (Yang Y. et al., 2015).

### Fungal Inhibition Assays

The effect of endophytic bacteria against hyphal growth was performed by using the dual-culture method (Yang L. et al., 2015). *Fusarium graminearum* PH-1 was grown on PDA at 28°C for 5 days, and then agar plugs excised and transferred into 100 ml CMC broth (Hou et al., 2002) and cultured at 160 rpm at 25°C for 5 days. Conidia were harvested by filtering the broth through Miracloth (EMD Millipore Corporation, Billerica, MA, United States), and the conidia were washed with sterile distilled water for 2–3 times and transferred into PDB with final concentration of 10^5^ spores/ml. Cells of strain YB-130 were grown for 10 h on LB agar at 37°C, washed from the plates with sterile distilled water and adjusted to 10^5^ cells per ml. After the bacteria were added to the PDB containing *F. graminearum* PH-1, the dual cultures were incubated shaking at 180 rpm at 28°C. PDB with pure culture of F. *graminearum* PH-1 was used as a control. The morphology of spores and hyphae were observed by using an Axio Imager A2 (Carl Zeiss, Oberkochen, DE, United States).

### Determination of DON Production and qPCR Quantification of Three DON Synthesis-Related Genes

After 5 days on PDA at 28°C, mycelial plugs of *F. graminearum* PH-1 were transferred into a flask with 100 mL CMC medium and incubated shaking at 160 rpm at 25°C for 5 days. The conidia were collected and washed by centrifugation, adjusted to 10^5^ conidia per ml in 4 ml trichothecene biosynthesis induction (TBI) medium (Menke et al., 2012). To this, YB-130 cells were obtained as previously described and added to a final concentration of 10^5^ cells per ml. The dual culture was incubated at 25°C for 72 h in the dark without shaking. TBI medium inoculated with *F. graminearum* PH-1 alone was used as a control. Levels of DON were determined using Deoxynivalenol-Z (DON-Z) Plate Kit (Beacon Analytical Systems, Saco, ME, United States) following the manufacturer’s instructions. After 72 h, the hyphae were isolated by filtering the medium through Miracloth (EMD Millipore Corporation, Billerica, MA, United States) and washing the hyphae twice with sterile distilled water. The hyphae was then immediately frozen in liquid nitrogen and stored at −80°C. Total RNA was extracted by TRIzol reagent (Invitrogen, Carlsbad, CA, United States) and reverse transcribed by PrimeScript RT reagent Kit with gDNA Eraser (Takara, Dalian, China) according to the manufacturer’s protocol for the SYBR Premix Ex Taq - kit. The expression of *tri3* (tri3-F: 5′-CTTGCAGGGATATCAAGAAATGTTACGA-3′ and tri3-R: 5′-CTCGCCTGTTGTAGTTCGCTTGATTT-3′), *tri5* (tri5-F: 5′-CCAGGAAACCCTACACTCGTCTAAG-3′ and tri5-R: 5′-TGGCCGCCTGCTCAAAGAAC-3 ′) and *tri8* (tri8-F: 5′-GCTACTTTGGACTCAATTCG-3′ and tri8-R: 5′-CATACTGTACCGCAAGTTCTG-3′) were calculated by the 2^–ΔΔ*Ct*^ method (Livak and Schmittgen, 2001) relative to that of ß-tubulin (βtub-F: 5′-GGTAACCAAATCGGTGCTGCTTTC-3′and βtub-R: 5′-GATTGACCGAAAACGAAGTTG-3′), which was included as the endogenous reference. Primer sequences were obtained from Lee et al. (2014) and Jiao et al. (2008), and PCR conditions were those of Lee et al. (2014). The experiment was replicated three times.

### Data Deposition

The raw PacBio sequencing genomic reads of strain YB-130 were deposited in NCBI Sequence Read Archive under the accession number of SRR11961844.

## Results

### Screening of Endophytes and Activity Against Plant Pathogenic Fungi

**FIGURE 1:**
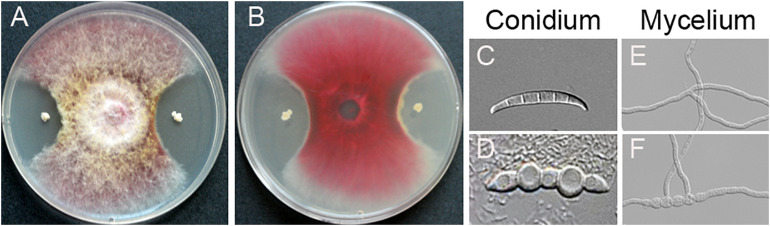
Colony morphology of *F. graminearum* co-cultivated with strain YB-130 showing upper **(A)** and lower **(B)** surface on PDA. Spore morphology of *F. graminearum* co-cultivated with **(D)** or without **(C)** strain YB-130. Hyphal morphology of *F. graminearum* co-cultivated with **(F)** or without **(E)** strain YB-130.

**FIGURE 2:**
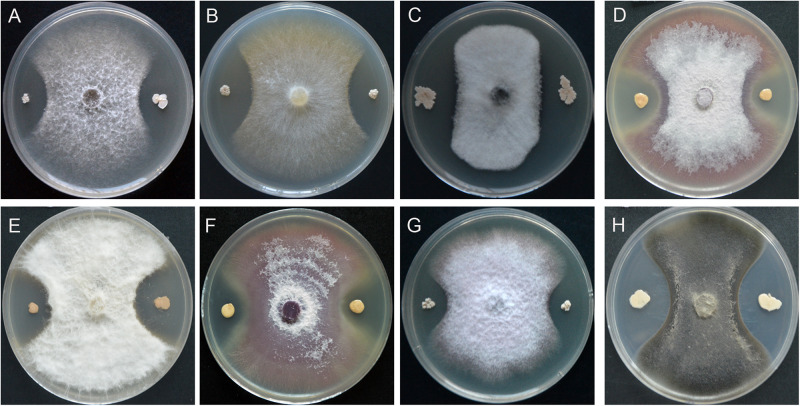
Effect of strain YB-130 on growth of eight plant pathogenic fungi. **(A)** *Gaeumannomyces graminis var. tritici*, **(B)** *Rhizoctonia cerealis*, **(C)** *Bipolaris sorokiniana*, **(D)** *Fusarium oxysporum f.sp. niveum*, **(E)** *Fusarium oxysporum f.sp. capsicum*, **(F)** *Fusarium oxysporum f.sp. cucumebrium*, **(G)** *Pythium myriotylum*, **(H)** *Cercospora apii*.

A total of 256 endophytic strains were isolated and purified from surface sterilized spikes of wheat with scab symptoms collected from the field. Dual cultures with *F. graminearum* PH-1 showed that 78 strains caused significant growth inhibition of *F. graminearum* PH-1 on PDA (data not shown). Of these, strain YB-130 displayed strong antagonistic activity against *F. graminearum* PH-1 (Figures 1A,B). Spores and hyphae of *F. graminearum* PH-1 were swollen in dual cultures with strain YB-130 compared to the control (Figures 1C–F). Culturing strain YB-130 with other eight plant pathogens, *Gaeumannomyces graminis* var. *tritici*, *Rhizoctonia cerealis*, *Bipolaris sorokiniana*, *Fusarium oxysporum* f.sp. *niveum*, *Fusarium oxysporum* f.sp. *capsicum*, *Fusarium oxysporum* f.sp. *cucumebrium*, *Pythium myriotylum*, and *Cercospora apii* showed significant inhibition of the growth of all the pathogens tested (Figure 2). This indicates that *B. velezensis* YB-130 exhibited a relatively broad spectrum against oomycetes, ascomycetes, and basidiomycetes.

**FIGURE 3:**
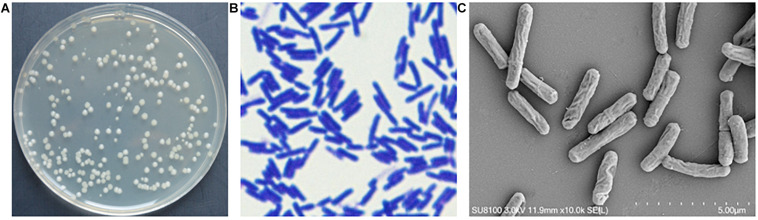
Morphology of strain YB-130. **(A)** Colony morphology of strain YB-130 on NA; **(B)** Gram staining of strain YB-130; **(C)** Scanning electron microscopy (SEM) images of strain YB-130.

On NA, strain YB-130 colonies were ivory white, non-transparent with a smooth surface (Figure 3A). Strain YB-130 was gram-positive (Figure 3B). SEM images showed that strain YB-130 cells were rod-shaped with an irregular wrinkled outer surface (Figure 3C).

### Genome Sequencing and Assembly of Strain YB-130

**FIGURE 4:**
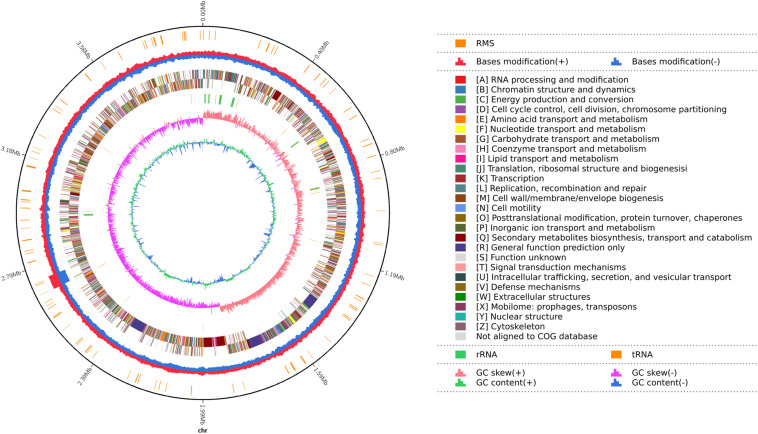
Map of the *B. velezensis* YB-130 genome. The distribution of circles from outwards to inwards are: ring 1 for genome size (black line); ring 2 for restriction modification system, for forward strand (red) and reverse strand (blue); ring 3 for COG classifications of protein-coding genes on the forward strand and reverse strand; ring 4 for the distribution of tRNAs (brown) and rRNAs (green); ring 5 for GC skew; ring 6 for GC content.

There was a total of 219,950 high-quality sequencing long reads from the DNA of strain YB-130 with a mean length of 10,829bp and a N50 of 13002 bp. Total base pairs was 2,381,982,861 bp with a 527.26X genome coverage, and the assembled genome was deposited at GenBank (Accession CP054562). The YB-130 genome consisted of a single circular chromosome of 3,980,767 bp with an average GC content of 46.46% (Figure 4). From three replicates of RNA of pure cultures of strain YB-130 grown for 24 h on NB, 50,894,066 paired-end reads were obtained. Alignment of the reads to the assembled genome sequence showed that for three replicates, 97.57, 98.52, and 97.70% of the reads were mapped to a unique location in the assembled YB-130 genome sequence with overall alignment rates of 98.96, 99.38, and 99.04%, respectively (unpublished data).

### Genome Annotation

The whole genome was predicted to have 4,105 protein-coding genes covering 89.20% of the genome with the average length of 865 bp, as well as 86 tRNAs and 27 rRNAs. Predicted protein sequence similarity searches against various databases with an *E*-value threshold of 1e-5 revealed that 4090 (99.63%), 3516 (85.65%), 3039 (74.03%), 2182 (53.15%), and 2209 (53.81%) protein-coding genes showed matches in the NR, Swiss-Prot, COG, KEGG and GO databases, respectively (Supplementary Table 1). In the NR database, 75.8% of the sequences were most similar to sequences of species within the *Bacillus subtilis* group. The remaining sequences have a significant similarity with sequences of mostly *Bacillus velezensis* (10.66%), followed by *Bacillus* sp. 5B6 (5.28%) and *Bacillus amyloliquefaciens* (4.22%).

### Comparative Genomics of *Bacillus* Species

**FIGURE 5:**
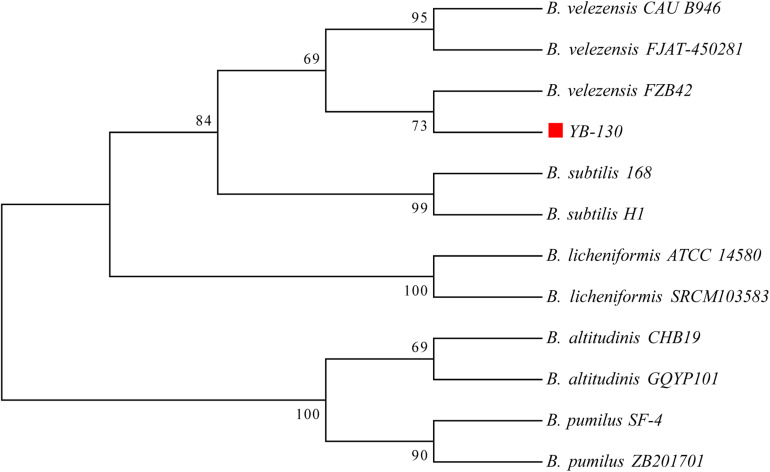
Phylogenetic tree of strain YB-130 and other 11 *Bacillus* species based on 16S rRNA sequence comparisons.

**FIGURE 6:**
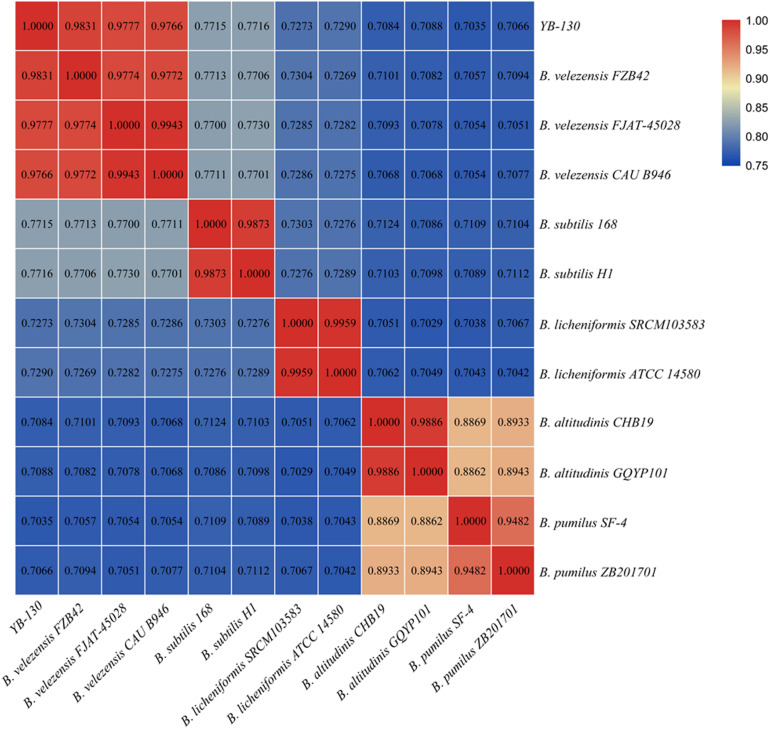
Heatmap of pairwise average nucleotide identity (ANI) values for whole genomes of strain YB-130 and 11 other *Bacillus* species.

A phylogenetic tree was constructed based on 16S rRNA gene sequences from strain YB-130 and 11 *Bacillus* species (Figure 5). Strain YB-130 was most closely related to *B. velezensis* FZB42 in a cluster with three other *B. velezensis* strains. Whole genome comparisons between strain YB-130 and other isolates showed that the highest ANI values (0.97 or higher) were only for isolates of *B. velezensis* (Figure 6). Genome matches *B. velezensis* FZB42, FJAT-450281 and CAU B946 were 98.31, 97.77, and 97.66%, respectively, which is higher than the cut-off of 95 to 96% for bacterial species (Richter and Rossello-Mora, 2009). Strain YB-130 and *B. velezensis* FZB42 had the maximum ANI value, which was in accordance with the clustering observed in the 16S rRNA tree. In contrast, ANI values between strain YB-130 and 8 other *Bacillus* species was much lower ranging from 70.35 to 77.16%. Therefore, the combination of 16S rRNA and whole genome sequence comparisons indicated that strain YB-130 should be classified as *B. velezensis*.

### Analysis of CAZymes in the YB-130 Genome

**FIGURE 7:**
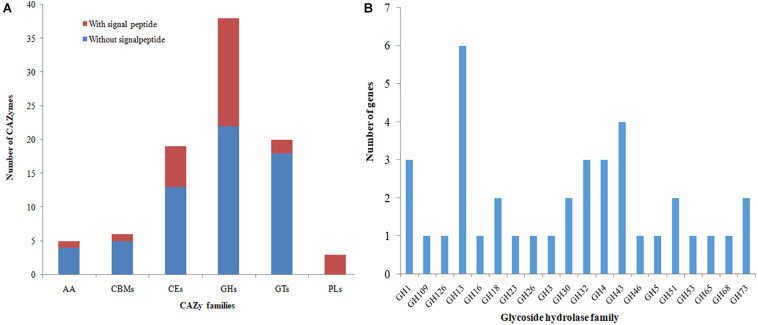
Distribution of CAZy families in the genome of strain YB-130. **(A)** Gene count distributions of carbohydrate-active enzyme (CAZy) families in the YB-130 genome; **(B)** Functional characterization of glycoside hydrolase family is based on the CAZy database.

A search for predicted proteins in the YB-130 genome for CAZymes showed that there were genes for 38 glycoside hydrolases (GHs), 20 glycosyltransferases (GTs), 3 polysaccharide lyases (PLs), 19 carbohydrate esterases (CEs), and 5 auxiliary activities (AAs), as well as 6 carbohydrate-binding modules (CBMs) that play a key role in promoting binding of enzymes with their substrates (Figure 7A). Three proteins, orf01878, orf01883, and orf03432, were classified as both GHs and CBMs. There were 27 (30.7%) of the CAZyme proteins with amino-terminal signal peptides for mediating export of proteins across the cytoplasmic membrane, indicating that they are secreted enzymes (Figure 7A and Supplementary Table 2). The largest group of secreted CAZymes were for the GHs with 16 out of 38 having signal peptides, while only one member of the AAs and CBMs was found with a signal peptide. The genome of *B. velezensis* YB-130 had 88 genes encoding for possible antifungal CAZymes in the GH families, such as chitinase (GH18), endoglucanase (GH51), and β-glucosidase (GH1), which have the potential to inhibit the growth of plant pathogens (Figure 7B). The distribution of CAZymes in the genome of strain YB-130 suggests that it poses a significant capacity to mediate antagonistic interaction with bacteria and fungi.

### Secondary Metabolic Potential of Strain YB-130

In the genome of strain YB-130, 12 gene clusters were found related to biosynthesis of secondary metabolites, accounting for 18.73% of the genes in the genome (Table 1). The putative gene clusters for secondary metabolites were three clusters encoding NRPS (non-ribosomal peptide synthetase), three clusters encoding transAT-PKS (*trans*-acyl transferase polyketide synthetase), two clusters for terpene biosynthesis, one cluster for T3PKS (Type III polyketide synthetase), one cluster for PKS-like (Type III polyketide-like synthetase), one cluster for lanthipeptide biosynthesis, one cluster with 100% similarity to bacilysin biosynthetic gene cluster. Among the three clusters encoding NRPS, two clusters had 100% similarity with known clusters for fengycin and bacillibactin synthesis and the other cluster had 82% similarity with known clusters for surfactin. The three clusters encoding transAT-PKS had 100% similarity with known clusters for the synthesis of bacillaene, difficidin, and macrolactin H, respectively.

**TABLE 1:** List of the putative gene clusters encoding for secondary metabolites by antiSMASH in strain YB-130 genome.

Gene Clusters	Types	Genome locations	Most similar known clusters	Similarity
Cluster 1	NRPS	323,590–387,567	surfactin	82%
Cluster 2	PKS-like	924,237–965,481	butirosin A/butirosin B	7%
Cluster 3	terpene	1,050,360–1,067,768		
Cluster 4	lanthipeptide	1,188,759–1,217,647		
Cluster 5	transAT-PKS	1,384,267–1,472,102	macrolactin H	100%
Cluster 6	transAT-PKS,T3PKS,transAT-PKS-like,NRPS	1,691,613–1,801,187	bacillaene	100%
Cluster 7	NRPS, transAT-PKS, betalactone	1,865,920–2,000,230	fengycin	100%
Cluster 8	Terpene	2,028,868–2,050,751		
Cluster 9	T3PKS	2,114,069–2,155,169		
Cluster 10	transAT-PKS-like, transAT-PKS	2,270,155–2,376,337	difficidin	100%
Cluster 11	NRPS, bacteriocin	3,052,028–3,103,819	bacillibactin	100%
Cluster 12	other	3,640,133–3,681,551	bacilysin	100%

Confirmation of the production and secretion of antimicrobial compounds by strain YB-130 was shown by cell-free extracts of the strain inhibiting *F. graminearum* PH-1 growth in petri dishes (Supplementary Figure 1). MALDI-TOF-MS was applied to detect surfactin and fengycin in cell-free extracts of strain YB-130, but only surfactin was detected (Supplementary Figure 2). There were molecular ion peaks (M + Na)^+^ for C_12_-C_15_ surfactin at m/z1016.6, 1030.6, 1044.6, and 1058.6, and ion peaks (M + K)^+^ for C_13_-C_17_ surfactin at m/z 1046.6, 1060.6, 1074.6, 1088.6, and 1102.6. Multiple molecular ion peaks for surfactin suggests the presence of varied lengths of fatty acid chains, which is consistent with that of previous reports for surfactin (Kowall et al., 1998; Koumoutsi et al., 2004).

Ten of the 12 gene clusters encoding for secondary metabolites in the genome of strain YB-130 were present in the genomes of other three *B. velezensis* strains (Supplementary Table 3). However, the cluster for the synthesis of lanthipeptide was only found in the strain YB-130 genome, which was missing the gene cluster for the synthesis of LAP (linear azoline-containing peptides) present in the *B. velezensis* FZB42, FJAT-450281 and CAU B946 genomes. These results indicate that antifungal spectrum produced by strain YB-130 is slightly different from other *B. velezensis* strains.

### Effect of YB-130 on DON Production and Gene Expression of DON Synthesis-Related Genes

**FIGURE 8:**
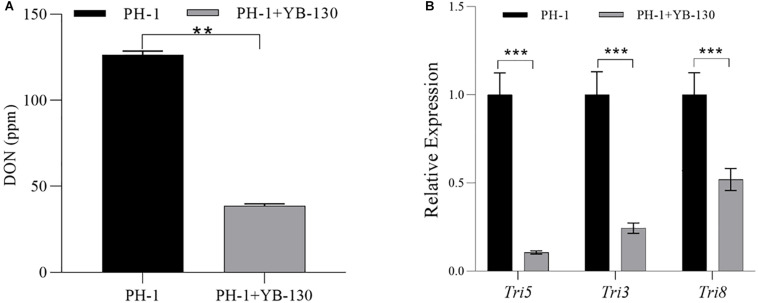
Effect of strain YB-130 on DON production and *tri* gene expression in *F. graminearum*. **(A)** DON production in *F. graminearum* PH-1 co-cultivated with or without strain YB-130. Mean DON value ± standard deviation (SD) obtained from three replicates for each sample (*N* = 3). **P < 0.01. A two-tailed *P* value was calculated between n *F. graminearum* PH-1 co-cultivated with or without strain YB-130 by *T*-test. **(B)** Expression of *tri5, tri3* and *tri8* in *F. graminearum* PH-1 co-cultivated with or without strain YB-130. Data were Mean ± standard deviation (SD) obtained from three replicates for each sample (*N* = 10). ***P < 0.001. Two-tailed *P* values were calculated between n *F. graminearum* PH-1 co-cultivated with or without strain YB-130 using *T*-test.

The DON concentration in co-cultures of *F. graminearum* PH-1 with strain YB-130 was significantly lower than with *F. graminearum* PH-1 alone (Figure 8A). By 72 hpi, pure cultures of *F. graminearum* PH-1 produced 125.3 ppm ± 0.5 ppm DON, which was significantly higher than the 38.5 ppm ± 0.3 ppm DON in the co-culture. Furthermore, expression of *tri5*, *tri3* and *tri8* were all significantly down-regulated relative to that of a constitutive control, ß-tubulin, in co-cultures of *F. graminearum* PH-1 with strain YB-130 (Figure 8B). The greatest reduction was for *tri5* showing a 10-fold down-regulation in the co-culture. The down-regulation by strain YB-130 was not due to growth inhibition of the fungus as the inclusion of ß-tubulin gene expression adjusted for the reduced growth of *F. graminearum* PH-1 in the co-cultures. Reduced DON biosynthesis-related gene expression may explain the reduced accumulation of DON when *F. graminearum* PH-1 was co-cultured with strain YB-130.

## Discussion

Endophytic microorganisms provide a promising strategy for management of plant pathogens (Syed Ab Rahman et al., 2018). In the past, several endophytic *Bacillus* strains, such as *B. subtilis* SG6, *B. velezensis* RC218, and *B. amyloliquefaciens* FNL13 had been tested for the control of wheat scab (Palazzini et al., 2007; Zhao et al., 2014; Baffoni et al., 2015). Inoculation of wheat with these showed about 72.6, 45, and 50% reductions in the levels of scab by *B. subtilis* SG6, *B. velezensis* RC218 and *B. amyloliquefaciens* FNL13, respectively (Palazzini et al., 2007; Zhao et al., 2014; Baffoni et al., 2015). This shows that such bacteria show promise as biocontrol agents. This study isolated and identified another *B. velezensis* isolate from a collection of 256 isolated endophytic strains exhibiting antagonism toward *F. graminearum* PH-1. *B. velezensis* YB-130 showed strongest very strong inhibitory activity to *F. graminearum* PH-1 as well as eight other crop pathogens *in vitro*. It was identified as *Bacillus velezensis* by morphological and 16S rDNA and whole-genome sequence analysis.

Other *Bacillus* isolates, such as *B. subtilis*, *B. amyloliquefaciens*, and *B. velezensis* had been found to inhibit spore morphology or mycelial growth of *F. graminearum* (Zhao et al., 2015; Jamal et al., 2017; Lee et al., 2017; Chen et al., 2018; Wang et al., 2020). Abnormally swollen spores and hyphae of plant pathogens were previously reported in dual cultures with endophytic bacteria (Lee et al., 2017; Saechow et al., 2018; He et al., 2019). In agreement with results of previous studies, morphological changes were observed by light microscopy analysis in our research and the micrographs showed that YB-130 could inhibit spore morphology and hyphal development of *F. graminearum* and lead to produce abnormally swollen spores and hyphae in dual culture antagonism assay. This could be due to the production of fungal cell wall degrading enzymes, which has been reported from a number of *Bacillus* species, including *Bacillus subtilis* AF 1 (Manjula and Podile, 2005), *Bacillus thuringiensis* C25 (Shrestha et al., 2015), *Bacillus cereus* 28-9 (Huang et al., 2005), and *Bacillus velezensis* ZJ20 (Xu et al., 2016).

The sequencing of the genome of *B. velezensis* YB-130 also revealed gene clusters encoding for several secondary metabolites. Among the ones observed, previous reports have shown that terpene (Gershenzon and Dudareva, 2007), lanthipeptide (McAuliffe et al., 2001), bacilysin (Wu et al., 2015), fengycin (Hanif et al., 2019), bacillibactin (Arguelles-Arias et al., 2009), bacillaene (Arguelles-Arias et al., 2009), difficidin (Wu et al., 2015), and macrolactin H (Ortiz and Sansinenea, 2020) have activity against filamentous fungi. The cluster for the synthesis of lanthipeptide was only found in strain YB-130 genome and not present in the other three *B. velezensis* genomes. Lanthipeptides with antimicrobial activity are called lantibiotics (McAuliffe et al., 2001). Lantibiotics produced by *Bacilli* exhibited antimicrobial activity against several plant pathogens including *Fusarium* species (Holtsmark et al., 2006; Palazzini et al., 2016; Xin et al., 2016). Considering the range of potentially antifungal secondary metabolites as well as potentially antifungal secreted CAZymes, it is not surprising that YB-130 significantly inhibited the growth of a wide range of plant pathogens. Future work is needed to determine which factors contribute the most to this activity.

Deoxynivalenol (DON) is a key virulence factor of *F. graminearum* that led to outbreaks of wheat scab under favorable weather conditions (Dweba et al., 2017), and many *tri* genes were responsible for its biosynthesis (Chen et al., 2019). A *Bacillus velezensis* strain isolated from maize soil could both inhibited hyphal growth of *F. graminearum* and reduced DON accumulation (Hanif et al., 2019). This study shows that another strain of the same species that is endophytic also shows the same properties. Hanif et al. (2019) did not examine how DON accumulation was reduced, but this study showed that inhibition of *tri* gene expression may be a mechanism. Future work will examine how broad ranged is the antimicrobial effects of YB-130 on *F. graminearum* using mass sequencing. However, this study laid the foundation for further deciphering the molecular mechanism of *B. velezensis* YB-130 on hyphal development and mycotoxin production of *F. graminearum*.

## Data Availability Statement

The datasets presented in this study can be found in online repositories. The names of the repository/repositories and accession number(s) can be found below: The assembled genome sequence of *B. velezensis* YB-130 was deposited in GenBank at https://www.ncbi.nlm.nih.gov/genbank/, Accession CP054562. The raw PacBio sequencing genomic reads and RNA-seq reads of strain YB-130 were deposited in NCBI Sequence Read Archive under the accession number of PRJNA638177.

## Author Contributions

LY, QW, and WX conceived the research and designed the experiments. WX, LZ, MX, JL, and CW performed the experiments and analyzed the data. WX prepared the manuscript draft. PG, JZ, and RS critically revised the manuscript. All authors approved the final version of the manuscript.

## Conflict of Interest

The authors declare that the research was conducted in the absence of any commercial or financial relationships that could be construed as a potential conflict of interest.

## Footnotes

1 http://www.cazy.org/

2 https://antismash.secondarymetabolites.org/

## Supplementary Material

Supplementary Figure 1 Cell-free extracts of *B. velezensis* YB-130 inhibit *F. graminearum* PH-1 in PDA. **(A)** *F. graminearum* PH-1 in PDA containing sterile distilled water in the Oxford cup; **(B)** *F. graminearum* PH-1 in PDA containing cell-free extracts of *B. velezensis* YB-130 in the Oxford cup; **(C)** *F. graminearum* PH-1 in PDA; **(D)** *F. graminearum* PH-1 in PDA containing 10%(v/v) cell-free extracts of *B. velezensis* YB-130.

Supplementary Figure 2 MALDI-TOF-MS analysis of surfactin produced by *B. velezensis* YB-130.
