# Supplementary figures and images for: Isolation, Identification, and Complete Genome Assembly of an Endophytic Bacillus velezensis YB-130, Potential Biocontrol Agent Against Fusarium graminearum
Source: Front Microbiol. 2020 Dec 3;11:598285. doi: 10.3389/fmicb.2020.598285 (PMC7744476; doi:10.3389/fmicb.2020.598285)

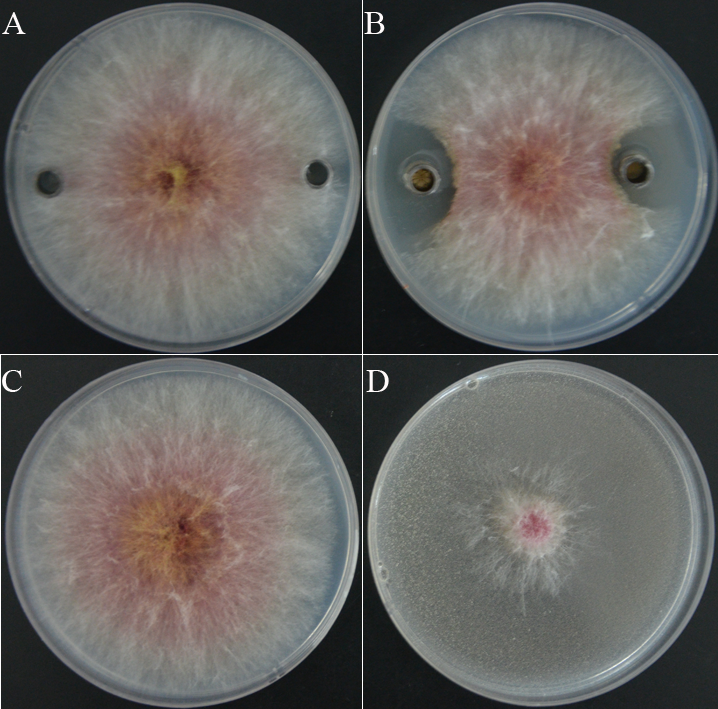

Supplement: Supplementary Figure 1 — Cell-free extracts of B. velezensis YB-130 inhibit F. graminearum PH-1 in PDA. (A) F. graminearum PH-1 in PDA containing sterile distilled water in the Oxford cup; (B) F. graminearum PH-1 in PDA containing cell-free extracts of B. velezensis YB-130 in the Oxford cup; (C) F. graminearum PH-1 in PDA; (D) F. graminearum PH-1 in PDA containing 10%(v/v) cell-free extracts of B. velezensis YB-130. [file Image_1.TIF]

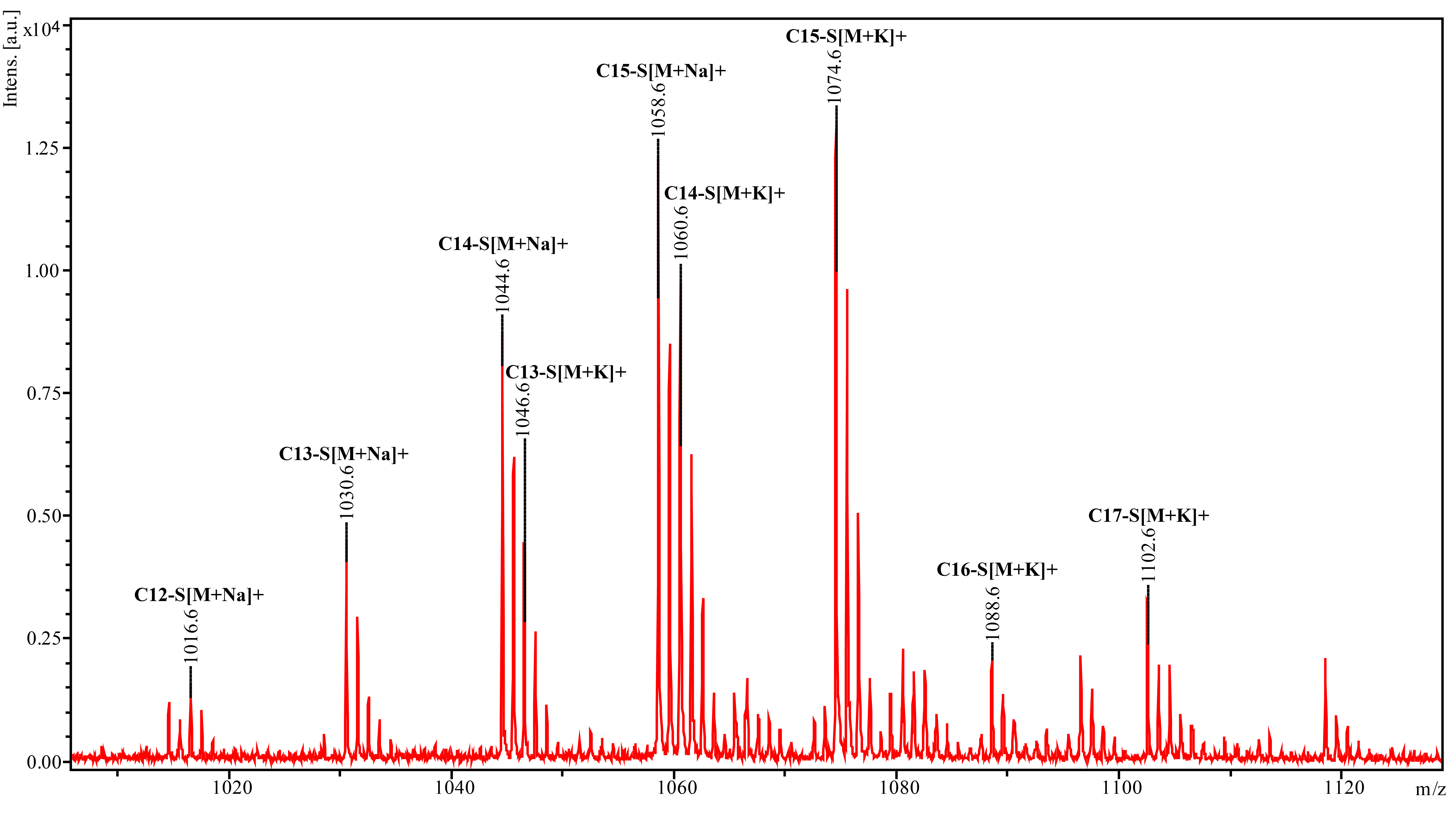

Supplement: Supplementary Figure 2 — MALDI-TOF-MS analysis of surfactin produced by B. velezensis YB-130. [file Image_2.TIF]
